# Protocol for a mixed-methods pilot study on the feasibility of TABATA training to alleviate moderate to severe psychological stress in Chinese middle school students

**DOI:** 10.3389/fpsyg.2026.1684786

**Published:** 2026-01-21

**Authors:** Li Hao, Arimi Fitri Mat Ludin, Mahadir Ahmad, Zhang Ben

**Affiliations:** 1 Center for Healthy Ageing and Wellness (HCARE), Faculty of Health Sciences, Universiti Kebangsaan Malaysia, Kuala Lumpur, Malaysia; 2 Nan Hang Secondary School, Nanjing, China; 3 Center for Community Health Studies (ReaCH), Faculty of Health Sciences, Universiti Kebangsaan Malaysia, Kuala Lumpur, Malaysia; 4 Military Fundamentals Department, Army Engineering University of PLA, Nanjing, China

**Keywords:** mixed-methods study, protocol, psychological stress, school students, TABATA training

## Abstract

Chinese secondary school students face increasingly complex psychological stress. Traditional interventions for adolescent psychological stress usually focus on psychological counseling or medication. However, the obvious drawbacks are potential side effects, limited attention to the root causes of mental health problems, and the risk of drug dependence. To address this, we developed a 20-min Tabata-style high intensity interval training module for this group. This program is designed to be efficient and time-saving, aiming to alleviate their psychological stress. This study will be executed in two phases: (1) Participants in the intervention group will undergo eight weeks of TABATA training, while those in the control group will not receive any intervention or participate in any interviews. (2) Participants for this phase will be selected from the RCT conducted in phase one. Individual interviews will be conducted with a subset of students who received the intervention, as well as with PE teachers who delivered the training. The results of this study will demonstrate the feasibility and acceptability of the TABATA training module we developed among Chinese middle school students experiencing moderate to severe psychological stress.

**Clinical trial registration:**

Study Protocol Registration details: China Clinical Trial registration number ChiCTR2500101576.

## 1 Introduction

Secondary school students are currently facing increasingly complex psychological stress (Pascoe et al., 2020). The intensification of social competition, the widespread phenomenon of educational involution, and the unique aspects of adolescent physical and mental development have made psychological stress a significant issue impacting their healthy growth and well-being (Wang et al., 2024). According to the World Health Organization, 2022 report, approximately 14% of adolescents aged 10 to 19 worldwide experience varying degrees of mental health issues. The primary triggers identified include academic pressure, family expectations, and social anxiety (Varzaneh et al., 2024).

Additionally, the Ministry of Education’s 2023 Blue Book on Youth Mental Health in China reveals that over 60% of secondary school students report experiencing moderate or higher levels of psychological stress, with this percentage showing a significant upward trend during critical promotional stages (Fu et al., 2021). This situation not only threatens the mental well-being of individuals but can also lead to a range of negative outcomes, such as academic burnout, strained interpersonal relationships, and crises of self-identity (Liu and Wang, 2024). Ultimately, these issues may have far-reaching implications for broader social development.

Traditional interventions for adolescent psychological stress typically focus on psychological counseling or pharmacotherapy. Psychological counseling offers several advantages: it addresses underlying issues, avoids drug-related side effects, and promotes self-awareness and improved interpersonal relationships (Tareke et al., 2023). However, this approach often requires longer treatment cycles, relies heavily on professional guidance, and can be more costly (Kiriakaki et al., 2022). In contrast, pharmacological therapies provide rapid symptom relief, are convenient to administer, and are widely applicable (Alorfi, 2023). Nevertheless, they have notable drawbacks, including potential side effects, limited focus on the root causes of mental health issues, and the risk of drug dependence (Sibley et al., 2023).

In recent years, a growing body of research has shown that moderate physical exercise can significantly reduce psychological stress (Mahindru et al., 2023). Through participation in sports activities, adolescents can effectively regulate their emotions, relieve anxiety and depression (Martinez et al., 2024), and find healthy outlets for emotional expression. Engaging in sports also helps build self-confidence and fosters resilience and courage in the face of challenges (Husain et al., 2024). On a social level, sports provide valuable opportunities for peer interaction and collaboration, thereby enhancing key social skills such as communication, teamwork, and leadership (Fernandez-Rio and Casey, 2021). Moreover, physical activity has been shown to improve cognitive functions in young people, including attention, memory, and decision-making (Latino and Tafuri, 2024). It also promotes the adoption of a healthy lifestyle, encouraging balanced nutrition and a regular daily routine.

Given the high academic pressure and limited time available for physical activity among Chinese secondary school students (Zhu et al., 2021), we have developed a time-efficient, TABATA-style high-intensity interval training (HIIT) module. TABATA training, recognized for its effectiveness in neuroendocrine regulation and minimal time requirements (Tabata, 2024), may be particularly well-suited for adolescents with demanding academic schedules. This method involves 20 s of high-intensity exercise followed by 10 s of rest, repeated for a total of eight cycles. It has been shown to enhance endorphin secretion and improve executive functioning (Tabata, 2022). However, the underlying mechanisms by which this training regulates psychological stress, as well as its feasibility and efficacy in adolescent populations, warrant further investigation. The primary objective of this study is to assess the feasibility and acceptability of the TABATA training module we have developed as an intervention to reduce psychological stress among Chinese adolescents. The secondary objectives are to evaluate changes in psychological stress level, physical fitness, academic performance, and quality of life.

## 2 Materials and methods

### 2.1 Study design

This is a two-stage, mixed-methods study comprising an initial feasibility randomized controlled trial (RCT), followed by a qualitative study. The reporting of this protocol will adhere to the SPIRIT guidelines. This study will be conducted in secondary schools in Nanjing City, Jiangsu Province, China. The research protocol received its primary ethical review and approval from the Ethics Committee of Nanjing Normal University (Approval Number: NNU202407008; Date: July 2024), which holds the principal oversight responsibility for ensuring full compliance with all applicable Chinese laws and regulations, including specific provisions for the protection of minors. Approval from the Universiti Kebangsaan Malaysia Research Ethics Committee (JEP-2024-544) was obtained subsequently by the project lead (a UKM doctoral candidate) for institutional academic purposes, contingent upon the primary approval from Nanjing Normal University. Consequently, all research activities will be carried out under the supervision and in accordance with the requirements of the Nanjing Normal University Ethics Committee. This study was also approved by Nan Hang Secondary School in October 2023. Upon verbal agreement, informed consent and assent forms will be obtained from parents and children, respectively.

### 2.2 Sample size

According to Whitehead et al. (2016) recommendations for pilot study sample sizes, to ensure the subsequent main trial achieves 90% power and a two-tailed 5% significance level, the sample size for each group in the pilot study should be set based on the expected effect size: a very small effect (0.1) corresponds to 75 participants, a small effect (0.2) to 25 participants, a medium effect (0.5) to 15 participants, and a large effect (0.8) to 10 participants. Furthermore, Dworkin (2012) noted in theory-based qualitative research that a sample size of 25–30 participants is typically required to achieve theoretical saturation and information redundancy. Integrating these recommendations from both statistical and qualitative research, this study sets the sample size per group at 25–30 participants. This aims to ensure sufficient statistical power while adequately capturing key experiences and feedback during the intervention process.

### 2.3 Eligibility criteria

The inclusion criteria are secondary school students in Nanjing, Jiangsu Province, aged 12 to 15 years, who are experiencing moderate to severe psychological stress as identified by the Chinese version of the DASS-21 questionnaire.

Exclusion criteria include students who are unable to participate in physical activity, have a diagnosed mental illness, or are unable to provide informed consent.

### 2.4 Recruitment and randomization

The participants in this study will be middle school students in Nanjing, Jiangsu Province, who are experiencing psychological stress. During the selection stage, students will complete the Chinese version of the DASS-21 questionnaire on a large scale through their school’s IT classes. Students with stress scores exceeding 19 will be identified as potential participants.

This study will employ a cluster randomized design. After obtaining informed consent, eligible participants will be grouped by their respective schools. All participants from one school will be assigned to the intervention group and receive the uniform intervention during the specified period. All participants from the other school will be assigned to the control group, maintaining their routine learning and daily activities without any intervention. During the evaluation process, outcome assessors will be blinded to ensure they remained unaware of the group assignments. This design is intended to minimize disruptions to regular teaching activities while ensuring participant privacy and safeguarding against potential stigmatization. Additionally, it helps prevent contamination between intervention and control groups, thereby preserving the study’s internal validity.

Given that the participants were minors experiencing psychological stress, strict measures were taken to ensure their privacy. All personal identifiers were removed from the transcribed data, and code numbers were used in place of participant names. Transcription files were encrypted and stored on a password-protected, stand-alone device, while physical materials were secured in a locked filing cabinet accessible only to the research team. All raw data were securely destroyed within 10 years of the study’s completion.

Participants in the intervention group will undergo 8 weeks of TABATA training, while those in the control group will not receive any intervention or participate in any interviews. Table 1 outlines the participant recruitment timeline, key milestones, and scheduled assessment activities. Stage 2 will involve the qualitative phase of the study. Participants for this phase will be selected from the intervention group in Stage 1. Individual interviews will be conducted with a subset of students who received the intervention, as well as with PE teachers who delivered the training. The aim of this phase is to evaluate the feasibility and acceptability of the TABATA training module.

**Table 1:** SPIRIT (Standard Protocol Items: Recommendations for Interventional Trials) schedule of enrolment, interventions, and assessment.

Timepoint	Study period										
	Enrolment	Allocation	Post-allocation								Close-out
	9/2025–10/2025	9/2025–10/2025	Week 1	Week 2	Week 3	Week 4	Week 5	Week 6	Week 7	Week 8	12/2025
Enrolment											
Screening	✓										
Eligibility screen	✓										
Informed consent	✓										
Allocation		✓									
Interventions											
Intervention A			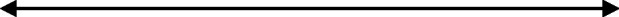								
Control			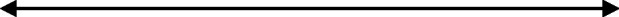								
Assessments											
DASS-21 Chinese version		✓				✓				✓	
Quality of life scale for children and adolescents (QLSCA) Chinese version		✓				✓				✓	
Physical fitness performance		✓				✓				✓	
Blood pressure		✓				✓				✓	

### 2.5 Interventions

#### 2.5.1 Randomized controlled trials

The TABATA training module is specifically designed for secondary school students experiencing psychological stress. Its aim is to reduce stress among them. This training method is characterized by its high efficiency and time-saving structure.

**Figure 1:**
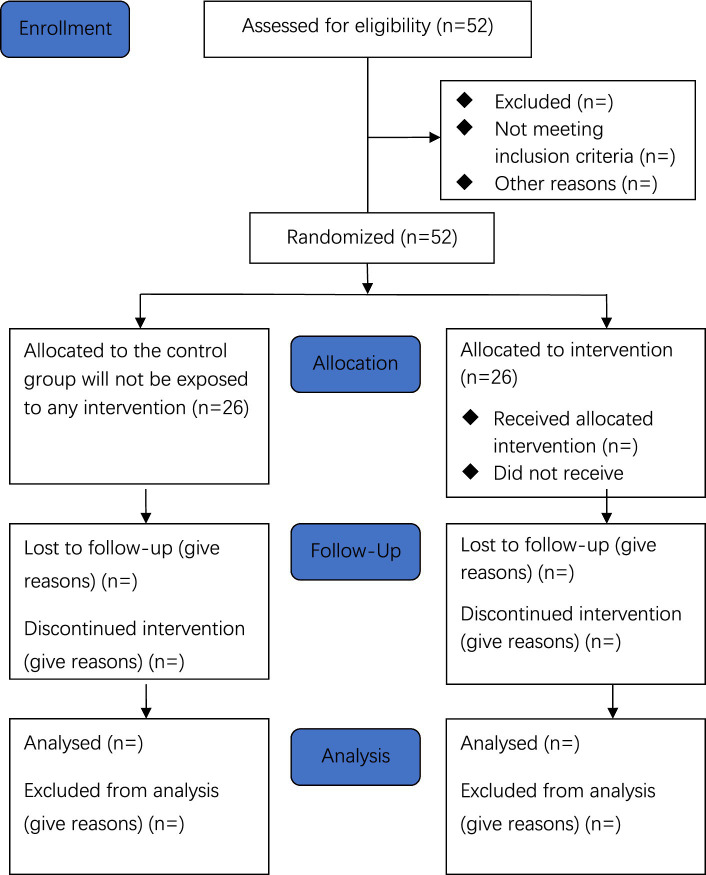
Consolidated standards of reporting trails 2010 flow diagram.

An eight-week intervention will be implemented, consisting of three sessions per week. This schedule is informed by an analysis of adolescents’ motivations, barriers, and preferences. The intervention will be delivered by physical education (PE) teachers at each participating school. A flowchart outlining the intervention process is presented in Figure 1.

The exercise intervention will be structured into three components:

- An 8-min warm-up jog
- An 8-min TABATA training session
- A 4-min relaxation and stretching cooling down

#### 2.5.2 Warm-up jog

The warm-up jog serves as a vital preparatory phase that promotes both physical and psychological readiness (Marakushyn et al., 2024). Light, sustained jogging gradually increases muscle temperature, enhancing the elasticity and flexibility of muscle fibers while improving the speed and strength of muscle contractions. It also boosts blood circulation, ensuring an adequate supply of oxygen and nutrients for the upcoming workout, and facilitates the removal of metabolic waste. These physiological effects help significantly reduce the risk of sports-related injuries such as muscle strains and joint sprains (Bushman, 2024).

In addition to its physical benefits, the warm-up jog stimulates the nervous system, improving reaction time and coordination, thereby enhancing focus and performance during the main workout. Psychologically, it helps reduce anxiety, build confidence, and mentally prepare students for the challenges ahead (Temaj et al., 2024).

#### 2.5.3 TABATA training

The TABATA training module has been tailored to the needs of secondary school students under psychological stress. To ensure accessibility and ease of implementation, the module consists of simple, easy to follow exercises. It includes eight different movements, performed in two rounds, with a total duration of 8 min. The detailed exercise routine is provided in Table 2.

**Table 2:** The content of TABATA training module.

Round 1	Movements	Round 2	Movements
0:00–0:20	Jumping jacks	0:00–0:20	Jumping jacks
0:20–0:30	Rest	0:20–0:30	Rest
0:30–0:50	High knees	0:30–0:50	High knees
0:50–1:00	Rest	0:50–1:00	Rest
1:00–1:20	Jumping lunges	1:00–1:20	Jumping lunges
1:20–1:30	Rest	1:20–1:30	Rest
1:30–1:50	Air squats	1:30–1:50	Air squats
1:50–2:00	Rest	1:50–2:00	Rest
2:00–2:20	Butt kicker	2:00–2:20	Butt kicker
2:20–2:30	Rest	2:20–2:30	Rest
2:30–2:50	Standing abs twist	2:30–2:50	Standing abs twist
2:50–3:00	Rest	2:50–3:00	Rest
3:00–3:20	Half Squat Jump	3:00–3:20	Half Squat Jump
3:20–3:30	Rest	3:20–3:30	Rest
3:30–3:50	Squat jump	3:30–3:50	Squat jump
3:50–4:00	Rest	3:50–4:00	Rest

To ensure consistency and safety of the intervention, physical education teachers participating in this study will receive systematically standardized training prior to the intervention. This training will be co-led by the principal investigator of the research team and a senior fitness coach, consisting of two sessions, each lasting 1 hour. Training will cover an overview of the overall research protocol; demonstration and correction methods for standard TABATA movements; intensity monitoring techniques based on heart rate and the Rating of Perceived Exertion (RPE) scale; safety guidelines; and adverse event recording procedures. Following training, teachers must pass a practical demonstration assessment to participate in the formal study. Additionally, teachers will receive a standardized set of TABATA training music tracks to ensure consistent movement tempo and rest intervals across all sessions. During the study implementation phase, the principal investigator will conduct random on-site observations to evaluate teachers’ adherence to the research protocol (including time management, intensity monitoring, and movement standardization), further ensuring intervention fidelity.

#### 2.5.4 Relaxation and stretching for cooling down

Relaxation stretching is an essential component of post-exercise recovery (Peake, 2019). It effectively reduces muscle tension and fatigue, enhances circulation to facilitate lactic acid removal, and minimizes post-exercise muscle soreness. Additionally, it improves flexibility and joint mobility, helping to prevent injuries caused by muscle stiffness and muscular imbalances (Loh et al., 2022).

Stretching also optimizes muscle contraction patterns, increases movement efficiency, and promotes smoother, more coordinated motor function, which contributes to improved overall athletic performance (Zvetkova et al., 2023). When combined with deep breathing and slow, controlled movements, relaxation stretching fosters mental relaxation, reduces post-exercise anxiety, and promotes a sense of calm. Furthermore, it supports blood and lymphatic circulation, aiding in nutrient delivery, waste elimination, and the recovery of muscles and tissues (Jermaina et al., 2022).

Accordingly, each TABATA session will conclude with a 4-min relaxation and stretching routine.

#### 2.5.5 Intensity monitoring

This study will employ a combined objective and subjective approach to monitor and evaluate exercise intensity. For objective monitoring, participants will wear heart rate monitoring wristbands (Huawei brand) during all TABATA training sessions to track real-time heart rate changes. For subjective assessment, participants will be instructed to self-report fatigue levels immediately after each session using a modified Borg RPE scale (0–10), with instructors conducting verbal interviews and recording responses.

#### 2.5.6 Suspension and exit

If a participant experiences paleness, dizziness, shortness of breath, chest pain, or any indication of severe discomfort, the training session will be immediately suspended for evaluation by the instructor and school nurse. If a participant is unable to perform a movement correctly due to technical inexperience or temporary fatigue, the instructor will provide a scaled-down alternative movement to ensure they can maintain target heart rate and engagement within safe parameters, rather than ceasing exercise entirely.

### 2.6 Attendance and adherence

Each training session will feature a paper attendance sheet where instructors will record student participation. Participants’ exercise adherence will be quantified through the following methods: (a) Actual exercise duration data exported from heart rate monitoring devices; (b) Instructors will document each student’s completion of TABATA sets; (c) RPE values collected after each training session. Absences or incomplete sessions will be noted with the reason.

### 2.7 Data collection instruments and outcome measures

The primary and secondary outcomes are defined in Tables 3, 4.

**Table 3:** The primary outcomes of the study.

Primary outcome measures	Definition	Success threshold
Recruitment rate	Proportion of all students who meet the eligibility criteria and agree to enter the study	>70%
Retention rate	Proportion of students that in the TABATA training group complete the 8-week intervention	>80%
Adherence rate	Actual number of times participated	>70%
Data missing rate	Overall missing data rate	<10%

**Table 4:** The secondary outcomes of the study.

Secondary outcome measures	Measurement indicators
Stress score	Changes in DASS-21 stress scores pre and post intervention
Quality of Life	Change of score on validated quality of life questionnaires QLSCA pre and post intervention
Physical fitness performance	Changes of score on Physical Fitness Test for Chinese Secondary School Students pre and post intervention
Academic performance	Changes in academic performance ranking pre and post intervention
Acceptability	Qualitative evidence generated from focus group discussions with participants

#### 2.7.1 The depression anxiety stress scale-21 (DASS-21), Chinese version

The DASS-21 is a concise yet reliable self-report instrument designed to assess the severity of negative emotional states, specifically depression, anxiety, and stress, over a given period (Cao et al., 2023).

The scale comprises 21 items, divided equally into three subscales, with each subscale containing seven items that evaluate symptoms related to depression, anxiety, and stress.

Stress subscale scores range from 0 to 34, with severity levels classified as follows: normal (0–14), mild (15–18), moderate (19–25), severe (26–33), and extremely severe (≥34). The Chinese version of the DASS-21 has been validated and demonstrates strong reliability and validity in assessing psychological distress among Chinese-speaking populations.

#### 2.7.2 Quality of life scale for children and adolescents (QLSCA)

The QLSCA is a multidimensional self-report instrument designed to assess the quality of life of primary and secondary school students aged 7 to 18. It evaluates four core domains: physical well-being, psychological well-being, social functioning, and living environment. Specific dimensions include physical health, mental health, social relationships, environmental conditions, and overall life satisfaction.

The QLSCA uses a four- or five-point Likert scale to capture responses, generating both an overall score and subscale scores for each dimension. These scores provide a comprehensive overview of a child’s or adolescent’s perceived quality of life. The Chinese version of the QLSCA has demonstrated strong reliability and validity in previous studies (Wu et al., 2006).

#### 2.7.3 Physical fitness performance

The Physical Fitness Test for Chinese Secondary School Students serves as a comprehensive tool to assess students’ overall physical fitness levels (Zhang et al., 2021).

It covers various aspects, including physical form (e.g., height and weight), physical function (e.g., lung capacity), and physical fitness (e.g., 50-meter sprint, seated forward bend, standing long jump, pull-ups/sit-ups, and endurance running). Test results will be converted into individual and total scores based on standardized scales and weightings. Students will then be classified into four performance categories excellent, good, pass, and fail according to their total scores for the academic year.

#### 2.7.4 Academic performance

The relationship between academic achievement and mental health is complex, characterized by mutual influence and interdependence. On one hand, academic pressure stemming from performance expectations can negatively affect students’ mental health, potentially leading to emotional issues such as anxiety and depression (Talley, 2024). On the other hand, a strong sense of academic achievement can enhance students’ self-esteem and self-confidence (Cuc and Cuc, 2019).

Moreover, students’ mental health directly influences their learning efficiency, emotional regulation, and overall academic performance (Usán Supervía and Quílez Robres, 2021). Given this bidirectional relationship, we will use the total scores from all subjects in the midterm and final examinations as indicators of academic achievement.

### 2.8 Interviews

The purpose of the qualitative phase is to explore participants’ and PE teachers’ perceptions and experiences with the TABATA training module. Based on previous study (Hoover et al., 2018), the interview questions will address various topics, including participation experiences, physical responses, psychological effects, curriculum design, and suggestions for improvement. The interview transcript is shown in Tables 5, 6.

**Table 5:** The students interview transcript template.

No.	Interview question
1.	Please describe your experience participating in the TABATA training course.
2.	What was the most memorable movement for you throughout the entire module? Why?
3.	During the training, what specific physical changes did you notice? (e.g., fatigue level, heart rate, amount of sweating, muscle sensation)
4.	What impact do you think the TABATA training has had on your physical fitness, endurance, or overall physical condition?
5.	How did you typically feel emotionally after participating in TABATA training? (e.g., excited, stressed, focused, bored).
6.	During or after the training, did you experience any changes in your sense of accomplishment, motivation, self-confidence, or willpower? Please describe specifically.
7.	Were there moments when you felt anxious, apprehensive, or wanted to give up? How did you cope with them?
8.	Do you think the current structure, duration, intensity, and movement arrangement of the TABATA training module are reasonable? Why?
9.	What are your thoughts on the music, the teacher’s demonstrations and explanations, and the monitoring bracelet?
10.	What do you think are the advantages and potential disadvantages of this training method, respectively?
11.	Would you be willing to recommend this course to classmates? Why or why not?
12.	Regarding safety, what areas do you think require special attention or need more guidance?
13.	Do you have any suggestions?

**Table 6:** The teachers interview transcript template.

No.	Interview question
1.	Please describe what it’s like to lead a TABATA training session.
2.	What burdens do you face while conducting TABATA training?
3.	How would you describe the overall engagement and emotional responses of the students toward the TABATA training?
4.	What impact do you believe this training program will have on students’ physical and mental fitness?
5.	Do you consider the current structure of the TABATA module (duration, intensity, exercise sequence) to be appropriate?
6.	How do you feel about the music used in the course and the monitoring devices? How do these elements impact teaching effectiveness and your workload?
7.	During implementation, which movements do you consider to pose higher security risks?
8.	What specific suggestions do you have for improving this intervention plan?

A semi-structured interview format will be adopted (Ruslin et al., 2022) in this study, allowing for in-depth exploration while maintaining a consistent framework across to facilitate in-depth exploration within a consistent framework. This phase will focus particularly on evaluating the acceptability and feasibility of the TABATA training module. Participants will from the intervention group and the physical education (PE) teachers who delivered it. To capture diverse perspectives, we aim to interview a minimum of 2 PE teacher and at least 20 students, ensuring representation of different roles within the program (Bekele and Ago, 2022). We will also strive to maintain a gender balance among student participants, targeting an approximate 1:1 male to female ratio to account for potential perceptual differences (Smith et al., 2018). This sampling approach is designed to ensure a meaningful variation in experiences and viewpoints.

The determination of data saturation will follow the methodological framework proposed by Francis (Francis et al., 2010). Specifically, data collection will commence with an initial set of 10 interviews. Following this, interviews will be conducted and analyzed in iterative batches of three. This process will continue until a complete batch yields no new themes or substantive information, at which point saturation will be considered achieved. This iterative approach ensures methodological rigor in meeting qualitative saturation standards while optimizing the efficient use of research resources.

Safeguarding the emotional well-being of adolescent participants, who experiencing psychological stress, is a central ethical priority. A protocol will be established for this purpose. First, written informed consent will be obtained from both participants and their guardians, detailing the interview’s purpose, potential risks, confidentiality, and the unconditional right to withdraw. These points will be verbally reaffirmed at the interview’s outset. Second, all interviewers will receive specialized training in engaging with vulnerable youth. They will be instructed to monitor participants for signs of stress throughout the session and to terminate the interview immediately upon such signs or the participant’s explicit request to stop. Finally, each interview will be designed to last approximately 10 min to minimize potential fatigue and anxiety, ensuring focus remains on the core topics.

### 2.9 Data analysis

The analysis will focus on obtaining descriptive statistics and confidence intervals for the variables. Participants will be characterized by means, standard deviations, and ranges for quantitative variables, as well as counts and proportions for categorical variables. Since this is a feasibility study primarily aimed at testing our ability to collect data, we will not use data interpolation to address any missing data. Based on the proposal for the pilot study, we have set the following feasibility goals: within 1 month, the intervention group and the control group will each recruit 20 to 30 participants; achieving a recruitment rate of over 70% in the TABATA intervention group; ensuring that more than 80% of students in the TABATA training group complete the 8-week intervention; having more than 70% of adherence rate; and maintaining an overall missing data rate of less than 10%.

All interviews will be recorded using an Apple mobile phone and transcribed verbatim, followed by manual proofreading using Flying Book of Notes software. To gain a comprehensive understanding of participants’ perceptions, an inductive analysis approach will be employed to identify broader themes (Azungah, 2018). Data will be analyzed using NVivo 12 qualitative analysis software to extract key themes related to feasibility and acceptability.

To enhance the credibility and reliability of the findings, researcher triangulation will be used to minimize researcher bias and strengthen the overall robustness of the study (Natow, 2020). First, one member of the research team will input the transcribed data into NVivo 12 and conduct initial coding in collaboration with another team member, merging codes to form preliminary themes. Next, two additional researchers will review the coding and refine the thematic structure for further integration.

Inter-rater reliability will be assessed by calculating the Kappa coefficient using NVivo 12. A Kappa value greater than 0.75 will be considered acceptable, indicating high consistency and data validity. Finally, all members of the research team will convene to finalize the themes related to the feasibility and acceptability of the TABATA training module.

### 2.10 Monitoring adverse events

Adverse events will be documented throughout the trial using a combination of participant self-reports and on-site observations by the physical education teacher administering the intervention. Each recorded event will include details such as the date, severity, time, location, duration, clinical measures taken, and outcomes.

## 3 Results

The research team submitted the trial application to the China Clinical Trial Registry and received approval, with registration number ChiCTR2500101576. Once the study is completed, we are committed to uploading the trial data to the Public Management Platform for Clinical Trials (http://www.medresman.org.cn) within 6 months to ensure openness, transparency and timely sharing of the study results.

## 4 Discussion

Chinese secondary school students experience intense academic competition, social pressure, and family expectations, which contribute to significant psychological stress (Zhou et al., 2023). While traditional psychological interventions have primarily focused on cognitive therapy (Ruiz, 2012), and some studies have explored the impact of exercise on mental health (Dunn and Jewell, 2010; Fossati et al., 2021), TABATA training has been less commonly used as an intervention. Unlike generic TABATA, the present study employed the Delphi method to develop a localized, modified model that incorporates stress-relieving goals. These include avoiding competitive elements in movement design (to reduce psychological stress) and shortening the duration of each session to 6–8 min (to accommodate the fragmented time between classes). As a result, this module represents an innovative approach.

However, it is important to recognize that, when applied in the school context, two major challenges arise: the feasibility and acceptability of implementation. This includes how to optimize training time and space within the constraints of a tight teaching schedule, ensure the adequacy and appropriateness of resources and facilities, and ensure there are enough qualified teachers to guide the training. Additionally, we must consider how to increase students’ interest and participation, gain teachers’ recognition and support, and overcome barriers related to cultural and social factors (Barnard and Henn, 2023). To address these challenges, further research is needed to gather multidimensional feedback, adjust the training program, and actively seek external resources such as policy support, funding, and professional training. These steps will be crucial in promoting the effective implementation of the TABATA training model in the school environment.

To address the issues outlined above, our research program adopts a mixed-methods approach (Almeida, 2018). This methodology combines both quantitative and qualitative research, allowing for methodological complementarity and providing more comprehensive and robust evidence to support the investigation of complex issues. Quantitatively, we will calculate and report the achievement of the following key indicators: recruitment rate (>70%), retention rate (>80%), intervention adherence rate (>70%), and data missing rate (<10%), to objectively measure the operability of the research process and the level of participant engagement. Qualitatively, we will conduct semi-structured interviews with teachers to systematically understand the compatibility of the course schedule and the actual workload of the teachers; simultaneously, we will conduct focus group interviews with students to gather in-depth information on their satisfaction with participation, perceived benefits, and the obstacles they face. These two approaches complement each other, forming a complete chain of evidence for assessing the feasibility of the intervention.

To ensure the accuracy of this study’s results, controlling potential biases is crucial. Therefore, we will take the following specific measures: First, to reduce selection bias, we will recruit potential participants through multiple channels and across a wide range of demographics, strictly adhering to established inclusion and exclusion criteria. Only individuals who meet all criteria and voluntarily sign informed consent forms will be invited to participate in the study, ensuring the representativeness and homogeneity of the selected sample. Second, to minimize attrition bias and improve participation and follow-up compliance, we will provide participants with standardized nutritional support weekly during the intervention period and, after the study concludes, present them with commemorative cultural and creative gifts as a token of appreciation for their full participation and task completion. Finally, all measurements will be conducted using uniformly calibrated equipment, and data will be collected using validated standardized recording forms, thereby minimizing expectation effects and measurement biases caused by differences in measurement tools or assessors.

## 5 Conclusion

We present the rationale and design concepts for a mixed-methods feasibility study of a self-developed TABATA training model aimed at alleviating psychological stress in Chinese secondary school students. The primary participants in the program are Chinese secondary school students and secondary school physical education teachers. The results and data from the program will be published 6 months after the completion of the trial.
